# Enhanced Diagnostic Precision: Assessing Tumor Differentiation in Head and Neck Squamous Cell Carcinoma Using Multi-Slice Spiral CT Texture Analysis

**DOI:** 10.3390/jcm13144038

**Published:** 2024-07-10

**Authors:** Lays Assolini Pinheiro de Oliveira, Diana Lorena Garcia Lopes, João Pedro Perez Gomes, Rafael Vinicius da Silveira, Daniel Vitor Aguiar Nozaki, Lana Ferreira Santos, Gabriela Castellano, Sérgio Lúcio Pereira de Castro Lopes, Andre Luiz Ferreira Costa

**Affiliations:** 1 Department of Anesthesiology, Oncology and Radiology, Faculty of Medical Sciences, University of Campinas (UNICAMP), Campinas 13084-971, SP, Brazil; lays_08@hotmail.com; 2 Postgraduate Program in Dentistry, Dentomaxillofacial Radiology and Imaging Laboratory, Department of Dentistry, Cruzeiro do Sul University (UNICSUL), São Paulo 01506-000, SP, Brazil; dilomagar@hotmail.com; 3 Department of Stomatology, Division of Oral Radiology, School of Dentistry, University of São Paulo (USP), São Paulo 05508-000, SP, Brazil; joao.pedro@usp.br; 4 Institute of Physics Gleb Wataghin, Universidade Estadual de Campinas (UNICAMP), Campinas 13084-971, SP, Brazil; r137382@dac.unicamp.br (R.V.d.S.); gabriela@unicamp.br (G.C.); 5 Brazilian Institute of Neuroscience and Neurotechnology (BRAINN), Campinas 13083-970, SP, Brazil; 6 School of Dentistry of Paulista Association of Dentists (FAOA), São Paulo 02010-000, SP, Brazil; daniel.odontofaoa@gmail.com; 7 Department of Diagnosis and Surgery, São José dos Campos School of Dentistry, São Paulo State University (UNESP), São José dos Campos, São Paulo 12245-000, SP, Brazil; lana.santos@unesp.br (L.F.S.); sergioluciolopes@gmail.com (S.L.P.d.C.L.)

**Keywords:** computed tomography, computer-assisted diagnosis, oral cancer, radiomics, tumor

## Abstract

This study explores the efficacy of texture analysis by using preoperative multi-slice spiral computed tomography (MSCT) to non-invasively determine the grade of cellular differentiation in head and neck squamous cell carcinoma (HNSCC). In a retrospective study, MSCT scans of patients with HNSCC were analyzed and classified based on its histological grade as moderately differentiated, well-differentiated, or poorly differentiated. The location of the tumor was categorized as either in the bone or in soft tissues. Segmentation of the lesion areas was conducted, followed by texture analysis. Eleven GLCM parameters across five different distances were calculated. Median values and correlations of texture parameters were examined in relation to tumor differentiation grade by using Spearman’s correlation coefficient and Kruskal–Wallis and Dunn tests. Forty-six patients were included, predominantly female (87%), with a mean age of 66.7 years. Texture analysis revealed significant parameter correlations with histopathological grades of tumor differentiation. The study identified no significant age correlation with tumor differentiation, which underscores the potential of texture analysis as an age-independent biomarker. The strong correlations between texture parameters and histopathological grades support the integration of this technique into the clinical decision-making process.

## 1. Introduction

Head and neck squamous cell carcinoma (HNSCC), which includes both oropharyngeal squamous cell carcinoma (OPSCC) and oral cavity squamous cell carcinoma (OCSCC), is a malignant tumor originating from keratinocytes within the squamous epithelium of the head and neck region [1,2]. It typically presents as cell groups arranged in cords and nests or as individual cells invading the connective tissue [3].

The degree of differentiation of tumor cells varies according to four parameters based on the Broders histological classification [4] as follows: grade 1 (0 to 25% of undifferentiated cells), grade 2 (25 to 50% of undifferentiated cells), grade 3 (50 to 75% of undifferentiated cells), and grade 4 (75 to 100% of undifferentiated cells). Meanwhile, there is a grading system for invasive margins, in which factors such as nuclear pleomorphism, invasion pattern, and degree of keratinization are taken into account [5].

The clinical diagnosis of a lesion is established based on a series of data obtained through anamnesis and physical examination. If neoplasia is suspected, a biopsy is mandatory to define the case through histopathological examination [6]. Imaging exams (e.g., computed tomography) help with diagnosis and, most importantly, evaluation of the extent of the tumor. The clinical examination, associated with biopsy and study of the lesion by computed tomography (CT), allows us to determine the tumor extent and define the treatment for the lesion [7,8,9,10].

However, despite significant advancements in the diagnosis and histopathological classification of HNSCC, there are still important gaps regarding the use of quantitative imaging techniques to assess tumor cell differentiation non-invasively [11,12]. Although cellular differentiation is a critical factor for tumor behavior and prognosis, reliable and non-invasive methodologies for its evaluation are still being developed [11]. Previous studies [13] emphasize the importance of cellular differentiation in determining tumor behavior, with poorly differentiated carcinomas showing a higher propensity for metastasis. Furthermore, its potential as a reliable biomarker, particularly in distinguishing between various grades of cellular differentiation, has not been extensively studied.

Considering the profound impact of the degree of cellular differentiation on prognosis, it becomes imperative to deeply investigate its significance, particularly in the context ofHNSCC. Recent evidence, as highlighted by Kademani et al. [14], underscores the critical role of cellular differentiation in determining tumor behavior, with poorly differentiated carcinomas demonstrating a heightened propensity for metastasis. This observation not only highlights the importance of precise differentiation but also of the urgency for reliable, new methodologies. Such predictive tools could revolutionize the clinical practice by enabling clinicians to anticipate disease aggressiveness and tailor treatment strategies, ultimately enhancing patient outcomes.

Texture analysis, a quantitative imaging technique, has emerged as a promising tool for characterizing tissue properties and identifying subtle histological features associated with tumor behavior [15,16,17,18]. By quantifying texture features of imaging modalities such as CT or magnetic resonance imaging (MRI), texture analysis can potentially enhance diagnostic accuracy and prognostic stratification of HNSCC [16,18].

Therefore, the aim of this study was to determine, through texture analysis, the grade of cellular differentiation in HNSC. By correlating texture features extracted from preoperative multi-slice spiral CT imaging with histopathological findings, and we aimed to establish a non-invasive, objective method for evaluating tumor differentiation, with implications for prognosis and treatment planning.

## 2. Materials and Methods

### 2.1. Patients

The study was conducted according to the guidelines of the Declaration of Helsinki and approved by the Institutional Review Board (or Ethics Committee) of Faculty of Medical Sciences at the University of Campinas (UNICAMP) (protocol code: 33377320.0.0000.5404; date of approval: 16 October 2020). All subjects provided a signed written informed consent form.

In this retrospective study, a search was conducted in our institution’s database in order to identify all patients with HNSCC according to the following inclusion criteria: histopathologically proven HNSCC, no radiotherapy or “making interpretation difficult” chemotherapy, and contrast-enhanced MSCT scans. Exclusion criteria were applied to patients with tumors of histological types other than OPSCC, images with artifacts hindering interpretation, neoplasia images acquired without intravenous contrast administration (iodine), and unclear medical records. HNSCC was histologically graded as moderately differentiated, well-differentiated, and poorly differentiated, whereas tumor location was categorized as either in the bone or in soft tissues.

The classification of tumor location in the bone or soft tissues was specifically designed to facilitate the image processing during texture analysis. This targeted approach not only streamlines the image processing workflow but also potentially increases the accuracy and reliability of the texture analysis. Furthermore, the separation into the bone or soft tissue categories acknowledges the distinct texture features between these two types of tissues [19].

Recognizing the biological and histopathological differences between OPSCC and OCSCC, including the differing role of human papillomavirus (HPV) infection, both types were included in the study. This decision was driven by the limited sample size and the retrospective nature of the study, as excluding OPSCC cases would have further reduced the statistical power of the analysis. Previous research, such as the study by Kuno et al. [20], has demonstrated the feasibility and relevance of including a mixed sample of HNSCC for texture analysis.

### 2.2. CT Image Acquisition

All patients underwent a 64-section contrast-enhanced MSCT scan (Aquilion TSX-101A model, Toshiba Medical Systems Corporation, Tokyo, Japan) preoperatively by using a multi-slice system and field of vision (FOV) of 320 mm, yielding slice thickness and reconstruction interval of 3 mm each and matrix size of 512 × 512. The scanner was operated at 120 kilovolts kVp and 400 mA.

### 2.3. Image Processing and Analysis

**Figure 1:**
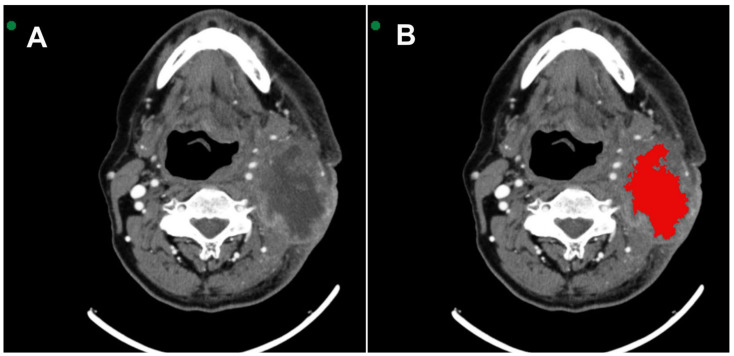
(**A**) Axial MSCT image shows squamous cell carcinoma located in the tonsillar pillar on the left side. (**B**) 3D VOI (red) segmentation process covering the lesion.

All images were acquired in Digital Imaging and Communications in Medicine (DICOM). MSCT scans were selected based on the identification of the largest area of the lesion. Two radiologists with 6 and 16 years of experience in interpreting MSCT scans were extensively involved in reviewing all the cases. They performed the segmentation with mutual agreement. The InVesalius^®^ software (www.cti.gov.br/invesalius, accessed on 5 July 2023) was used for the initial image processing. Once all the lesions were segmented and similar areas identified, the DICOM files were converted into the NIfTI format by using the InVesalius^®^ software toolbox [21]. Figure 1 shows the segmentation process before calculating the texture parameter values.

### 2.4. Texture Analysis

In this study, we assessed eleven gray-level co-occurrence matrix (GLCM) parameters from each segmented lesion volume by using texture analysis in the MATLAB software (MathWorks, Natick, MA, USA, www.mathworks.com, accessed on 19 July 2023). The GLCM is a matrix showing how many times a pair of pixel values appear at a specific distance and angle. The GLCM is used for capturing second-order statistical texture parameters such as contrast, correlation, entropy of difference, entropy, homogeneity, sum average, sum entropy, sum variance, uniformity, variance of difference, and variance. The mean value of the texture parameters was estimated on a volume of interest (VOI) basis. Hence, we opted for 128 levels as being the most suitable balance between GLCM shortage and amount of information extracted [22]. Previous studies have shown successful results by using Haralick parameters [21,23,24,25]. The number of slices used for GLCM calculation within the manually segmented VOI depending on the specific VOI being analyzed [22]. The texture parameters were assessed at five varying distances (q1, q2, q3, q4, and q5) and calculated similarly to the study by Gomes et al. [21].

### 2.5. Statistical Analysis

Spearman’s correlation coefficient was used to assess the correlation between distances of the same texture analysis parameter. Tumor differentiation grades were compared by using the Kruskal–Wallis test, followed by the Dunn test (numerical variables) or the Chi-Square test (categorical variables). Statistical analysis was conducted by using the software R, version 3.6.0 (The R Foundation for Statistical Computing, Vienna, Austria), with a significance level set at 5%.

## 3. Results

After applying the criteria for inclusion and exclusion, 45 patients were included. There was a female predominance (87%), and the mean age was 66.7 years (range: 33–93 years). The results showed that five (11.1%) patients had a moderately differentiated tumor in the bones, seven (15.6%) had well-differentiated tumor in the soft tissues, 30 (66.7%) had moderately differentiated tumor in the soft tissues, and three (6.7%) had poorly differentiated tumor in the soft tissues.

Texture analysis with eleven parameters at five different distances generated a large number of variables, which significantly increased the chance of type I error. Therefore, the variables were reduced by using the mean of the five distances. Before calculating the mean of the five distances for each parameter, the Spearman’s correlation coefficient between the distances was calculated and assessed. As a high correlation was observed in nine out of the eleven parameters, the mean value between the distances was used. In the correlation texture parameter, the distance 5 did not show a high correlation with the other distances. Therefore, this parameter was evaluated in two ways: the mean from 1 to 4 and 5 considered separately. A similar situation occurred with the texture parameter difference of entropy, where the mean of distances from 2 to 5 was used, and distance 1 was evaluated separately.

**Figure 2:**
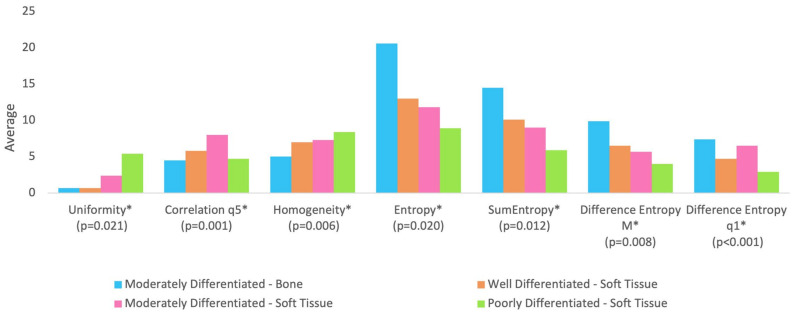
The groups are statistically different at 5% significance level regarding parameters followed by asterisk (*). All parameters were rescaled to be inserted into the graph in order to avoid distorting the scale for the other parameters.

Table 1 shows the median of age and texture parameters according to tumor differentiation grade groups. Table 2 shows the comparison between tumor differentiation grade in relation to age and texture analysis. It is observed that no statistically significant difference was found between the groups in relation to age (*p*-value = 0.725). Figure 2 shows the tumor differentiation grade groups significant for texture parameters.

With regard to texture analysis, the observed results are as follows:

- Group BM (moderately differentiated tumor in bone) showed lower correlation compared to Group SM (moderately differentiated tumor in soft tissue);
- Group BM (moderately differentiated tumor in bone) showed lower uniformity compared to Group SM (moderately differentiated tumor in soft tissue) and Group SP (poorly differentiated tumor in soft tissue);
- Group BM (moderately differentiated tumor in bone) showed lower homogeneity compared to Group SM (moderately differentiated tumor in soft tissue) and Group SP (poorly differentiated tumor in soft tissue);
- Group BM (moderately differentiated tumor in bone) presented higher entropy and sum of entropy compared to Group SM (moderately differentiated tumor in soft tissue) and Group SP (poorly differentiated tumor in soft tissue);
- Group BM (moderately differentiated tumor in bone) presented higher entropy of difference (mean of distances from 2 to 5) compared to Group SM (moderately differentiated tumor in soft tissue) and Group SP (poorly differentiated tumor in soft tissue);
- Group SW (well-differentiated tumor in soft tissue) and Group SP (poorly differentiated tumor in soft tissue) showed lower entropy of difference (distance 1) compared to Group BM (moderately differentiated tumor in bone) and Group SM (moderately differentiated tumor in soft tissue).

## 4. Discussion

The objective of this study was to assess the degree of cellular differentiation in HNSCC by relating texture parameters derived from preoperative MSCT scans with histopathological features. We endeavored to devise a non-invasive, objective technique for determining tumor differentiation. This approach has implications for prognosis and treatment planning, while also considering the location of the lesion in soft and bone tissues.

It is observed that the parameter of correlation indicated a statistically significant difference (*p* = 0.001) in the Group MD (moderately differentiated tumor in hard tissue) compared to Group SM (moderately differentiated tumor in soft tissue) and Group SP (poorly differentiated tumor in soft tissue), indicating lower values in Group BM. The parameter of correlation assesses the degree of interdependence between image pixels and higher values, which indicates a greater interdependence between pixels [26], that is, the uniformity of the tissue content represented by the pixel values would be greater. This indicates that there is no variation in tissue types comprising the region, with a consequently less complex internal pattern [27,28]. Thus, this finding means that tumors in hard tissue (BM) are less uniform than those in soft tissue, either poorly or moderately differentiated.

The parameters of uniformity and homogeneity obtained by texture analysis, which were statistically significant in Group SW compared to Groups SM and MP, corroborate what was previously indicated (i.e., correlation) for this same group. This reinforces that texture image values of patients with moderately differentiated tumor in bone indicate greater tissue organization [29].

Considering the parameters of entropy (i.e., entropy, entropy of difference, and sum of entropy), it can be observed that they all presented statistically significant differences between the Groups BM, SM, and SP, with the former having higher values than the others. These are complementary parameters and indicate disorganization of image pixels. In the previously cited study by Costa et al. [30], entropy was directly related to image disorganization, with lower values indicating a greater lack of uniformity in the tissue and a greater variation in shades of gray (i.e., internal components of the tissue), which often correlates with a higher degree of malignancy. This reflects the complexity and heterogeneity often seen in more aggressive tumors.

Moreover, the difference in entropy parameters between the groups further underscores the potential of texture analysis in discriminating between different degrees of tumor differentiation [31,32]. Specifically, higher entropy values in Group BM (moderately differentiated tumor in bone) suggest a more chaotic and less predictable tissue structure compared to the soft tissue groups. This aligns with the notion that bone tumors, due to their denser and more complex structure, may exhibit more pronounced texture irregularities in the images [33], which can be captured through advanced analytical techniques such as those used in this study.

The findings of this study support the use of texture analysis in the context of medical imaging, particularly in enhancing our understanding of tumor biology and behavior without the need for invasive procedures [16,34]. By incorporating a detailed set of textural parameters, this study adds to the current knowledge and suggests further areas for exploration in the use of imaging analysis in oncology. For instance, a recent study demonstrated the efficacy of automated analysis of CT scans by employing textural features and machine learning techniques, achieving high accuracy in their applications, highlighting the potential of texture analysis in diverse medical contexts [35].

It is important to acknowledge the limitations of this study. The sample size, although adequate for a preliminary analysis, is relatively small and could affect the generalizability of the findings. Additionally, our sample included both OPSCC and OCSCC cases. Although we recognize the significant biological and histopathological differences between these two types of squamous cell carcinoma, including the differing role of HPV infection, we opted to include both to maintain a robust sample size. Excluding the small number of OPSCC cases would have further reduced the statistical power of our analysis. This approach is supported by the study conducted by Kuno et al. [20], which demonstrated the feasibility and relevance of using CT texture analysis across different subtypes of head and neck squamous cell carcinoma. Their research showed that CT texture features could significantly predict treatment outcomes, underscoring the potential of texture analysis to provide valuable insights even when diverse tumor subtypes are included in the study population. However, future studies should aim to separate these groups or control for these differences more explicitly to validate and expand upon our findings, ensuring a more nuanced understanding of how texture features correlate with tumor differentiation and patient prognosis.

Another limitation is the gender distribution of our sample, which was predominantly female (87%). This contrasts with the typical gender distribution in head and neck cancers, where males are usually more affected. The convenience sampling method used in this retrospective study resulted in a gender imbalance. This could influence the generalizability of our results, and future studies should aim for a more balanced gender distribution to ensure more representative findings

Further research involving larger cohorts and multiple cancer types would be valuable in validating and expanding our findings. Additionally, future studies should consider including HPV status and its potential impact on the analyzed texture parameters, especially in OPSCC cases. We have an ongoing prospective study that aims to address these points by incorporating a larger sample size and including cross-validation to further validate our findings and examine the benefits of including both OPSCC and OCSCC.

## 5. Conclusions

In conclusion, this study highlights the potential of texture analysis of preoperative MSCT images as a non-invasive method to assess tumor differentiation in HNSCC. The significant correlations found between texture parameters and histopathological features suggest that this approach could serve as a valuable tool in the clinical setting, aiding in the prognosis and tailoring of treatment strategies for patients with different tumor types. As medical imaging technology and analytical methods continue to evolve, the integration of texture analysis into clinical practice could enhance the precision and effectiveness of cancer diagnosis and treatment planning.

## Figures and Tables

**Table 1:** Median of age and texture parameters according to tumor differentiation.

Texture Parameters	Moderately Differentiated Bone (BM) (N = 5)	Well Differentiated Soft Tissue (SW) (N = 7)	Moderately Differentiated Soft Tissue (SM) (N = 30)	Poorly Differentiated Soft Tissue (SP) (N = 3)
Age	63.0 [59.0;85.0]	70.0 [59.0;89.0]	65.5 [33.0;93.0]	67.0 [63.0;73.0]
**Uniformity**	**0.06 [0.01;0.18]**	**0.18 [0.04;0.32]**	**0.20 [0.02;0.69]**	**0.69 [0.14;0.78]**
Contrast	76.8 [14.9;271]	24.8 [0.71;49.9]	9.63 [0.40;166]	0.97 [0.22;37.6]
Correlation M	0.75 [0.50;0.76]	0.86 [0.40;0.91]	0.73 [0.27;0.96]	0.63 [0.47;0.85]
**Correlation q5**	**0.50 [0.21;0.62]**	**0.76 [0.14;0.82]**	**0.84 [0.07;0.94]**	**0.40 [0.28;0.73]**
Variance	147 [24.2;243]	80.3 [2.67;125]	18.6 [0.47;279]	0.87 [0.27;109]
**Homogeneity**	**0.49 [0.36;0.64]**	**0.71 [0.54;0.88]**	**0.75 [0.39;0.91]**	**0.93 [0.65;0.93]**
**Entropy**	**2.12 [1.55;2.40]**	**1.29 [0.77;1.74]**	**1.08 [0.39;2.66]**	**0.39 [0.32;1.96]**
SumAverage	74.4 [59.0;126]	77.7 [58.4;131]	80.7 [24.5;137]	94.7 [77.7;98.2]
SumVariance	570 [82.0;702]	276 [9.98;473]	62.8 [1.19;951]	2.50 [0.88;399]
**SumEntropy**	**1.52 [1.15;1.63]**	**1.04 [0.68;1.26]**	**0.82 [0.33;1.80]**	**0.33 [0.26;1.17]**
DifferenceVariance	55.7 [6.81;212]	20.5 [0.61;40.4]	9.65 [0.35;141]	0.92 [0.20;31.9]
**DifferenceEntropy M**	**0.98 [0.79;1.19]**	**0.68 [0.31;0.84]**	**0.54 [0.24;1.22]**	**0.21 [0.21;0.78]**
**DifferenceEntropy q1**	0.71 [0.61;0.90]	0.50 [0.19;0.61]	0.64 [0.60;0.71]	0.19 [0.12;0.55]

CorrelationM = average of distances 1 to 4; DifferenceEntropyM = average of distances 2 to 5. Bold indicates significant *p* value.

**Table 2:** Comparison of tumor differentiation grade in relation to age and texture analysis.

Texture Parameters	Moderately Differentiated Bone (BM) (N = 5)	Well Differentiated Soft Tissue (SW) (N = 7)	Moderately Differentiated Soft Tissue (SM) (N = 30)	Poorly Differentiated Soft Tissue (SP) (N = 3)	*p*-Value
Age	67.0 (10.3)	71.1 (9.01)	65.5 (13.4)	67.7 (5.03)	0.725
**Uniformity**	**0.07 (0.07)**	**0.18 (0.10)**	**0.24 (0.16)**	**0.54 (0.35)**	**0.021**
Contrast	99.4 (104)	22.2 (16.5)	23.6 (39.5)	12.9 (21.4)	0.062
Correlation M	0.69 (0.11)	0.73 (0.21)	0.70 (0.20)	0.65 (0.19)	0.807
**Correlation q5**	**0.45 (0.15)**	**0.58 (0.28)**	**0.80 (0.16)**	**0.47 (0.23)**	**0.001**
Variance	127 (96.5)	59.0 (51.4)	52.1 (75.0)	36.7 (62.6)	0.123
**Homogeneity**	**0.50 (0.11)**	**0.70 (0.11)**	**0.73 (0.12)**	**0.84 (0.16)**	**0.006**
**Entropy**	**2.06 (0.34)**	**1.30 (0.36)**	**1.18 (0.53)**	**0.89 (0.93)**	**0.020**
SumAverage	84.7 (27.3)	83.6 (22.9)	85.3 (26.2)	90.2 (11.0)	0.836
SumVariance	445 (274)	214 (193)	184 (265)	134 (229)	0.092
**SumEntropy**	**1.45 (0.19)**	**1.01 (0.25)**	**0.90 (0.36)**	**0.59 (0.51)**	**0.012**
DifferenceVariance	75.6 (82.2)	18.1 (13.2)	19.7 (31.8)	11.0 (18.1)	0.096
**DifferenceEntropy M**	**0.99 (0.15)**	**0.65 (0.18)**	**0.57 (0.24)**	**0.40 (0.33)**	**0.008**
**DifferenceEntropy q1**	**0.74 (0.14)**	**0.47 (0.14)**	**0.65 (0.03)**	**0.29 (0.23)**	**<0.001**

CorrelationM = average distances 1 to 4; DifferenceEntropyM = average of distances 2 to 5. Bold indicates significant *p* value.

## Data Availability

The datasets generated and/or analyzed during the current study are available from the corresponding author upon reasonable request.
